# Correlation of end tidal carbon dioxide, amplitude spectrum area, and coronary perfusion pressure in a porcine model of cardiac arrest

**DOI:** 10.14814/phy2.13401

**Published:** 2017-09-12

**Authors:** Nicolas Segal, Anja K Metzger, Johanna C. Moore, Laura India, Michael C. Lick, Paul S. Berger, Wanchun Tang, David G. Benditt, Keith G. Lurie

**Affiliations:** ^1^ Department of Emergency Medicine University of Minnesota Medical School Minneapolis Minnesota; ^2^ ZOLL Medical Minneapolis Minnesota; ^3^ Department of Emergency Medicine Hennepin County Medical Center Minneapolis Minnesota; ^4^ Minnesota Medical Research Foundation Minneapolis Minnesota; ^5^ Department of Emergency Medicine Virginia Commonwealth University Richmond Virginia; ^6^ Cardiovascular Division Department of Medicine University of Minnesota Minneapolis Minnesota

**Keywords:** Carbon Dioxide/*analysis, cardiopulmonary resuscitation, electrocardiography/*methods, electrocardiography/methods/*statistics & numerical data, ventricular fibrillation

## Abstract

Amplitude Spectrum Area (AMSA) values during ventricular fibrillation (VF) correlate with myocardial energy stores and predict defibrillation success. By contrast, end tidal CO_2_ (ETCO2) values provide a noninvasive assessment of coronary perfusion pressure and myocardial perfusion during cardiopulmonary resuscitation (CPR). Given the importance of the timing of defibrillation shock delivery on clinical outcome, we tested the hypothesis that AMSA and ETCO2 correlate with each other and can be used interchangably to correlate with myocardial perfusion in an animal laboratory preclinical, randomized, prospective investigation. After 6 min of untreated VF, 12 female pigs (32 ± 1 Kg), isoflurane anesthetized pigs received sequentially 3 min periods of standard (S) CPR, S‐CPR+ an impedance threshold device (ITD), and then active compression decompression (ACD) + ITD CPR. Hemodynamic, AMSA, and ETCO2 measurements were made with each method of CPR. The Spearman correlation and Friedman tests were used to compare hemodynamic parameters. ETCO2, AMSA, coronary perfusion pressure, cerebral perfusion pressure were lowest with STD CPR, increased with STD CPR + ITD and highest with ACD CPR + ITD. Further analysis demonstrated a positive correlation between AMSA and ETCO2 (*r* = 0.37, *P* = 0.025) and between AMSA and key hemodynamic parameters (*P* < 0.05). This study established a moderate positive correlation between ETCO2 and AMSA. These findings provide the physiological basis for developing and testing a novel noninvasive method that utilizes either ETCO2 alone or the combination of ETCO2 and AMSA to predict when defibrillation might be successful.

## Introduction

The International Consensus on Cardiopulmonary Resuscitation 2010 recommends delivering a defibrillation shock every 2 min during treatment of cardiac arrest (Jacobs et al. 2010). These defibrillator shocks can be either life saving or cause significant harm, depending upon the timing of the shock and the status of the myocardium. Myocardial injury due to defibrillation is related to the severity of postresuscitation global myocardial dysfunction (Xie et al. 1997). In addition, interruptions in precordial compressions reduce coronary perfusion pressure (CoPP) which may compromise the success of the shocks, especially after prolonged cardiac arrest (Paradis et al. 1990).

To limit the number of unnecessary shocks and interruptions in precordial compressions, ventricular fibrillation (VF) waveform analysis has been established to predict the success of defibrillation at any given time. AMSA values during ventricular fibrillation (VF) correlate with coronary perfusion pressure/myocardial energy stores and predict more precisely defibrillation success (Povoas et al. 2002; Neurauter et al. 2008; Ristagno et al. 2008, 2013, 2015; He et al. 2016). Several different analysis methods have been developed and the most efficient of these methodologies is to examine the amplitude spectrum area (AMSA) values (Povoas et al. 2002; Neurauter et al. 2008; Ristagno et al. 2008). The technique to determine AMSA values however is generally not instantaneous due to the need to sequentially sample and filter a large amount of electrocardiographic data and then perform multiple calculations. At present, AMSA is not recommended for routine use in the guideline for defibrillation management in adult cardiac arrest in the clinical setting in or out‐of‐hospital (Jacobs et al. 2010). It is anticipated that as AMSA algorithms are incorporated into newer defibrillators that there may be a future opportunity to use this noninvasive assessment tool to help optimize the timing of defibrillation during cardiac arrest and CPR.

In contrast with AMSA, end tidal CO2 (ETCO2) measurements are commonly made during CPR. Noninvasive measurement of ETCO2 has been shown to correlate with coronary perfusion pressure and myocardial perfusion during CPR (Sanders et al. 1985; Weil et al. 1985). A threshold ETCO2 value has been correlated in animals and humans to predict outcomes. However, ETCO2 is not currently used to guide when defibrillation success could be most likely. Being able to predict shock success with ETCO2 would be an improvement of resuscitation protocol.

Given the importance of the timing of defibrillation shock delivery on clinical outcome, we tested the hypothesis that AMSA and ETCO2 correlate with each other and can therefore be used interchangeably to correlate with myocardial perfusion. Specifically, this study examined three CPR methods, each generating a different level of perfusion, to establish the association between ETCO2 and AMSA over a range of coronary perfusion pressure values. A positive correlation could then be used as a way to provide additional support for more widespread use of ETCO2 to help guide defibrillation therapy and CPR in general.

## Materials and Methods

### Experimental preparation

This study was approved by the Institutional Animal Care and Use Committee of the Minneapolis Medical Research Foundation (MMRF) of Hennepin County Medical Center. All animal care was compliant with current DHHS guidelines (*Guidelines for the Care and Use of Laboratory Animals*, 8th Edition, National Research Council, 2011), Animal Welfare Act regulations (*9 CFR Chapter 1, Subchapter A*) and Good Laboratory Practice for Nonclinical Laboratory Study under the US Code of Federal Regulations title 21. All studies were performed by a qualified, trained, and experienced research team. A licensed and board certified veterinarian (DACLAM) assured the protocols were performed in accordance with all applicable local, state, and federal requirements in the MMRF facility which is AAALAC International, Inc. accredited.

Twelve female farm pigs (32 ± 1 kg) pigs (domestic crossbreed) were fasted overnight. They were sedated with 10 mL (100 mg/mL) of intramuscular ketamine HCl (Ketaset, Fort Dodge Animal Health, Fort Dodge, IA). An intravenous bolus of propofol (PropoFlo, Abbott Laboratories, North Chicago, Il) (2–3 mg/kg) was given via a lateral ear vein and then infused at a rate of 160–200 *μ*g/(kg min) for the remainder of the preparatory phase. A 7.5 mm cuffed French endotracheal tube was inserted and positive pressure, volume control ventilation with a tidal volume of 10 mL/kg of room air was delivered through the tube with a NarkoMed 4A (North American Drager) ventilator. The respiratory rate was adjusted (average 12 ± 2 bpm) to keep oxygen saturation above 95% and ETCO_2_ between 38 and 42 mmHg.

While in a ventral recumbent position, an intracranial bolt was inserted into the animal's parietal lobe to measure intracranial pressure using a 3.5 French micromanometer pressure transducer (Miko‐Tip Transducer, Millar Instruments, Inc., Houston, TX) as previously described (Burnett et al. 2012). Animals were then placed supine. The left femoral artery and left external jugular vein were cannulated using a modified Seldinger percutaneous technique. Central aortic blood pressures were measured continuously via a micromanometer‐tipped Millar catheter placed in the chest cavity at the level of origin of the thoracic descending aorta. Central venous blood pressures were measured via a micromanometer‐tipped Millar catheter placed in the superior vena cava, approximately 2 cm above the right atrium. Central venous pressures were maintained between 4 and 6 mmHg during the preparatory phase. Carotid artery blood flows were measured using a bidirectional Doppler flow probe surrounding the internal carotid artery (Transonic Systems, Ithaca, NY). Surface ECG was also monitored continuously. A thermometer was placed in the rectum and body temperature maintained with a heating blanket between 37.0.0° and 38.0°C. All data were digitized using a computer data analysis program (BIOPAC MP 150, BIOPAC Systems Inc., CA). ETCO_2_, tidal volume and arterial oxygen saturation were recorded with a CO_2_SMO Plus (Novametrix Medical Systems, Wallingford, CT).

### Experimental design

After the preparatory phase, animals were positioned for CPR and prearrest hemodynamic variables were measured. Ventricular fibrillation (VF) was induced in the anesthetized animal with application of a 50 Hz, 7.5 V AC electrical current through an electrophysiology catheter to the endocardial surface of the right ventricle. Propofol anesthesia was discontinued. After 6 min of untreated cardiac arrest, mechanical CPR via a pneumatic piston attached to a compression pad was initiated. Chest compressions were performed with a rate of 100/min and a depth of 25% of the anteroposterior diameter as previously described (Lurie et al. 1995; Schultz et al. 2012). All animals were ventilated during CPR with supplemental oxygen (2 L/min) with a bag‐valve resuscitator at a compression to ventilation ratio of 10:1 and a tidal volume of 10 mL/kg. Three sequential CPR epochs were performed for a total of 9 min: 3 min of conventional closed chest or standard (STD) CPR, 3 min of STD CPR + impedance threshold device (ITD) (ResQPOD^TM^, Zoll, Roseville, MN), and 3 min of active compression decompression (ACD) (ResQPUMP™, Zoll) CPR + ITD. The transition from one method of CPR to the next was made in an uninterrupted manner. STD and ACD CPR were performed using an automated compression decompression device as previously described (Lurie et al. 1995). After the 9 min of CPR, epinephrine (40 *μ*g/Kg) was administered intravenously and 1 min later the pigs were defibrillated with up to 3 additional sequential 200 J transthoracic biphasic shocks. Following successful resuscitation the intravenous propofol was resumed and one hour later animals were euthanized with a bolus intravenous injection of 10 mol/L KCl (30 mg/Kg).

### Data analysis

The electrocardiographic (ECG) signal was sampled at 300 Hz and stored in 1.6 sec increments such that each 4 s wavelet was processed at intervals of 1.6 sec. The ECG signal was filtered between 3 and 30 Hz to minimize low‐frequency artifacts produced by precordial compression and to exclude the electrical interference of ambient noise at frequencies greater than 48 Hz. Analog ECG signals were digitized and converted from a time domain to a frequency domain by fast Fourier transformation via a computer data analysis program (BIOPAC). Utilizing MATLAB 5.1 software (Mathworks Inc., Natick, MA), the sum of individual amplitudes and frequencies resulted in the amplitude spectrum area (AMSA) (Povoas et al. 2002). This method to calculate AMSA was develop in the same sine model of cardiac arrest that we used during this study (Povoas et al. 2002). Power spectrums for the VF waveform were generated the same way.

The mean AMSA values for each pig for each intervention was used for the analysis. The mean values for all hemodynamic parameters extracted from multiple 4 sec intervals obtained contemporaneously with the AMSA data were measured and used for future analysis. All values with a non‐normal distribution are expressed as median (25–75 percentiles). A Friedman statistical test was conducted to analyze ETCO2, AMSA, the calculated coronary and cerebral perfusion pressure, aortic systolic, diastolic and mean pressure, right atrial pressure, and intracranial pressure during the three CPR methods. Coronary perfusion pressures were determined by the difference between the diastolic aortic pressure and diastolic right atrial pressure during each CPR intervention. Cerebral perfusion pressures were determined by taking the difference between the aortic pressure and the intracranial pressure. Spearman correlation and Friedman tests were used to analyze the correlation between the different hemodynamic parameters. A Bland and Altman assessment was used to compare ETCO2 and AMSA values with a range of agreement defined as mean bias ±1.96 SD*. P* < 0.05 were considered statistically significant. Statistical analyses were performed with SPSS^®^ Statistics 17.0 (IBM Corporation, Somers, NY).

## Results

There were significant differences in the ETCO2, AMSA, coronary perfusion pressure, cerebral perfusion pressure, systolic aortic pressure, mean aortic pressure, mean right atrial pressure, and mean intracranial pressure based upon the method of CPR used. Key perfusion parameters were lowest with STD CPR, increased with STD CPR + ITD and were highest with ACD CPR + ITD (Table 1).

**Table 1:** Median (25; 75 percentile); hemodynamic parameter using the three different cardiopulmonary resuscitation techniques

	ETCO2	AMSA	Ao sys	Ao dia	Ao mean	RA mean	ICP mean	CePP	CoPP	CBF
STD CPR	5.7 (4.5; 7.9)^a^	31.1 (26.9; 39.4)^a^	30.7 (28.3; 35.2)^a^	9.5 (8.4; 12.1)	20.2 (18.2; 22.4)^a^	12.6 (11.4; 15.7)^a^	18.3 (14.8; 22)^a^	2.8 (‐1.4; 8.1)^a^	8.4 (6.1; 10)^a^	25 (14; 36)
STD CPR + ITD	15.2 (13.9; 18.4)^b^	39.7 (29.9; 45.8)^b^	37.7 (33; 64.7)^b^	10.5 (8.9; 19.4)	22.9 (22; 39.9)^b^	15.5 (13.4; 27.5)^b^	19.6 (16.2; 23.7)	5.7 (3.2; 14.8)^b^	10.6 (7.9; 13.3)^b^	24 (14; 36)
ACD CPR + ITD	20.5 (16.5; 21.5)^c^	45.5 (31.5; 50.8)^c^	41.9 (37; 59.9)	11.5 (7.6; 18)	25.9 (23.7; 34.1)	16.6 (14.1; 22.3)	18.4 (15.2; 22.9)^c^	7.6 (4.9; 17)^c^	13.3 (7.9; 19.7)^c^	27 (19; 45)

Ao sys, systolic aortic pressure; Ao dia, diastolic aortic pressure; RA, right atrial pressure; ICP , intracranial pressure; CePP, cerebral perfusion pressure; CoPP , coronary perfusion pressure; ETCO2, end tidal CO_2_ (mmHg); AMSA, amplitude spectral area (mV‐Hz); CBF, carotid blood flow (ml/min).

a *P* = 0.001 STD CPR < STD CPR + ITD < ACD CPR + ITD (Friedman statistical test).

b *P* < 0.05 for difference between STD and STD + ITD.

c *P* < 0.05 for difference between STD + ITD and ACD CPR + ITD (Wilcoxon test).

**Figure 1:**
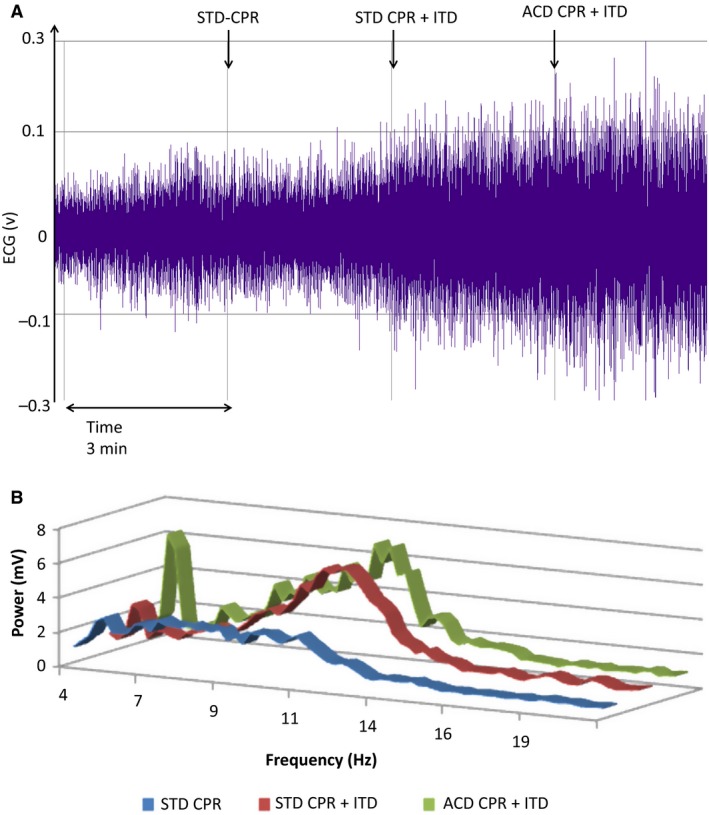
(A) ECG signal amplitude before fast Fourier transformation (same animal as B). STD, standard, ITD, impedance threshold device, ACD, active compression decompression. (B) power spectrum for the VF waveform based upon the methods of CPR. There was a pronounced increase in the high‐frequency signal in this representative study with the progression from STD CPR to ACD CPR + ITD. STD, standard; ITD, impedance threshold device; ACD, active compression decompression.

The power spectrum for the VF waveform was calculated using the raw VF waveform signal. The raw VF amplitude also changed significantly based upon the method of CPR (Fig. 1A). Figure 1B shows the power spectrum from a representative animal during each of the 3 methods of CPR. There was an increase in the absolute VF amplitude and in the high‐frequency signal during the progression change from STD CPR to STD CPR +ITD to ACD CPR + ITD. The respective median values for all animals are also shown in Table 1: the AMSA increased from 31.1 with STD CPR to 45.5 with ACD CPR + ITD.

**Figure 2:**
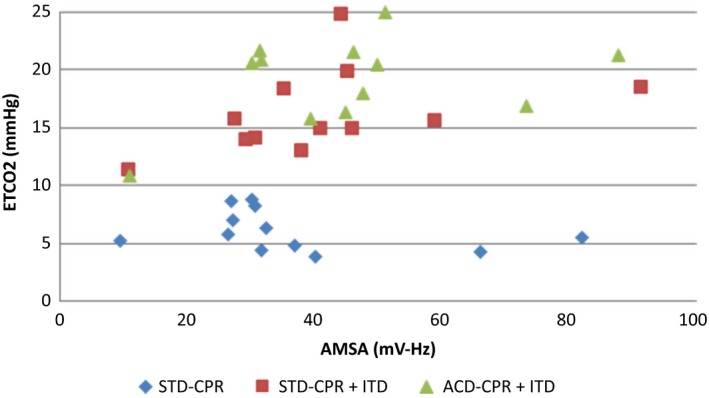
Spearman Correlation (*P* = 0.025; Rank=0.37) graphic; ETCO2, end tidal CO_2_ (mmHg); AMSA, amplitude spectral area (mV‐Hz); STD, standard; ITD, impedance threshold device; ACD, active compression decompression.

**Figure 3:**
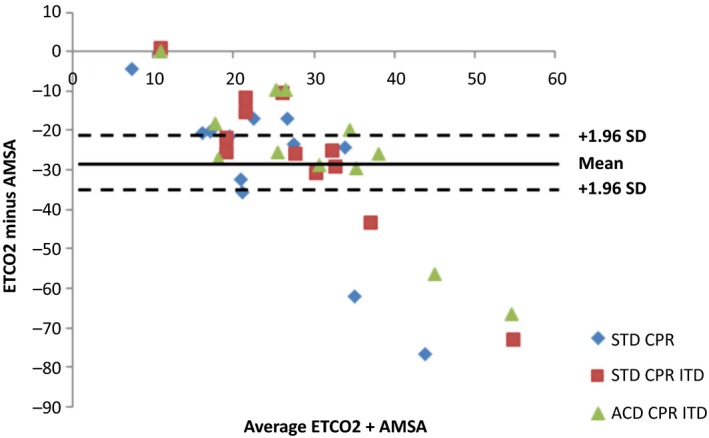
Bland and Altman graphic. ETCO2, end tidal CO_2_; AMSA, amplitude spectral area; STD, standard; ITD, impedance threshold device; ACD, active compression decompression.

The key demonstration of this study shows a significant correlation between AMSA and ETCO2 using the Spearman rank test (*r* = 0.37, *P* = 0.025) and a Spearman correlation between AMSA and key hemodynamic parameters (coronary perfusion pressure, cerebral perfusion pressure, aortic systolic, diastolic and mean pressure) (*P* < 0.05) (Fig. 2 and Table 2). The Bland‐Altman analysis indicated the 95% limits of agreement between AMSA and ETCO ranged from −21.4 to −33.5 (Fig. 3). These study results indicate a moderate association between AMSA and ETCO2.

**Table 2:** Correlation between the different hemodynamic parameter, SR and p, correlation is significant

SR p	AMSA	Ao sys	Ao dia	Ao mean	CePP	CoPP	CBF
ETCO2	0.374^a^ 0.025	0.709^a^ <0.001	0.315 0.061	0.723^a^ <0.001	0.526^a^ 0.001	0.340^a^ 0.043	0.295 0.08
AMSA		0.541^a^ 0.001	0.487^a^ 0.003	0.612^a^ <0.001	0.217 0.203	0.185 0.281	0.639^a^ <0.001
Ao sys			0.611^a^ <0.001	0.953^a^ <0.001	0.825^a^ <0.001	0.514^a^ 0.001	0.567^a^ <0.001
Ao dia				0.709^a^ <0.001	0.499^a^ 0.002	0.514^a^ 0.001	0.411^a^ 0.006
Ao mean					0.77^a^ <0.001	0.575^a^ <0.001	0.597^a^ <0.001
CePP						0.503^a^ 0.002	0.373^a^ <0.001

SR, Spearman rank; Ao sys, systolic aortic pressure; Ao dia, diastolic aortic pressure; Ao, aortic pressure; RA, right atrial pressure; ICP, intracranial pressure; CePP, cerebral perfusion pressure; CoPP, Coronary perfusion pressure; ETCO2, end tidal CO_2_ (mmHg); AMSA , amplitude spectral area (mV‐Hz); CBF, Mean carotid blood flow (mL/min).

a Correlation is significant.

All but one animal could not be resuscitated. The maximal AMSA and ETCO2 values in this single animal remained low, 10.9 and 11.3, respectively, even with ACD CPR + ITD.

## Discussion

Over the past decade, research has focused on finding a noninvasive method to predict the success of defibrillation with the hope of having a substantial impact on the survival outcome of patients. AMSA has been reported to provide an 86% positive and an 85% negative predictive value, respectively, for a threshold value at 21 mV × Hz (Povoas and Bisera 2000). However AMSA values can be difficult to calculate in real time and are not routinely use for defibrillation management in adult cardiac arrest in the clinical setting in or out‐of‐hospital (Jacobs et al. 2010). By contrast, continuous ETCO2 waveforms are readily obtainable, can be rapidly analyzed, and measurement of ETCO2 is highly recommended in CPR Guidelines. Using three different methods of CPR that have been previously demonstrated to consistently vary vital organ perfusion levels, (Burnett et al. 2012; Lurie et al. 2016) this study demonstrated for the first time a significant correlation and statistical agreement between ETCO2 and AMSA.

The concept of using ECG to guide CPR and defibrillation was first demonstrated by Weaver et al. who determined VF voltage was dependent on the length of time between initial collapse, start of basic life support, and the delay in the arrival of paramedics (Weaver et al. 1985). Similar to coronary perfusion pressure, several studies have indicated ECG to be a quantitative predictor of the success of CPR and,(Noc et al. 1994) is strongly associated with outcome survival rates.(Weaver et al. 1985) Amplification of VF voltage during cardiac resuscitation corresponds to an increase in myocardial perfusion and favorable changes in myocardial energy metabolism. In 2002, Povoas et al. demonstrated an AMSA value of 21 mV Hz or greater predicted restoration of a perfusion rhythm after defibrillation with a negative predictive value of 0.96 and a positive predictive value of 0.78, which is currently the best predictor of return of spontaneous circulation (Povoas et al. 2002). Several additional studies have confirmed this through comparable results (Neurauter et al. 2008; Ristagno et al. 2008).

ETCO2 has also served as a diagnostic tool during CPR, in a manner similar to ECG, as a reflection of cardiac output and blood flow (Weil et al. 1985; Gudipati et al. 1988). It is routinely measured in critically ill patients inside and outside the hospital. In animal models, increases in ETCO2 correspondingly result in successful resuscitation whereas those animals which fail to respond to resuscitation efforts have a lower ETCO2 (Trevino et al. 1985). A number of studies have determined that comparable to AMSA, ETCO2 is correlated with coronary perfusion pressure and myocardial perfusion in cardiac arrest (Sanders et al. 1985; von Planta et al. 1989). Accordingly, ETCO2 can be used to prognosticate survival outcome during CPR and immediately identifies cardiac arrest and restoration of spontaneous circulation (von Planta et al. 1989). These results have been confirmed in several human studies (Falk et al. 1988; Hatlestad 2004). ETCO2 has also been correlated with perfusion/microcirculation, (Callaham and Barton 1990) internal carotid blood flow and cerebral blood flow (Lewis et al. 1992).

Building upon prior studies, the objective of the current study was to determine if changes in ETCO2 based upon the level of circulation during CPR would correlate with changes in AMSA: if both ETCO2 and AMSA were found to correlate with coronary perfusion pressure and other key parameters reflective of coronary perfusion then a threshold ETCO2 level might be useful to predict when to defibrillate a patient in VF. The primary intent of the study was not to identify a critical threshold ETCO2 value that would predict defibrillation success in the pig model used. However, with a limited number of animals we did identify a threshold window for potential defibrillation success which was between 9 and 18 mmHg. That is, with ETCO2 values of less than 9 mmHg the chances for successful defibrillation is likely to be extremely low and with values of greater than 18 mmHg the chance for successful defibrillation are high. Our results are consistent with previously identified literature values (von Planta et al. 1989; Sanders et al. 1989; Callaham and Barton 1990; Cantineau et al. 1996; Levine et al. 1997; Grmec and Klemen 2001). Based upon the current results and the results of others, it seems reasonable to design an experiment in animals and to gather defibrillation success outcome data in humans to better determine the threshold ETCO2 value that is predictive of defibrillation success. Further, based upon the current study it is possible that a combination of an ETCO2 value and an AMSA value may have a higher sensitivity and specificity and be more rapidly achievable in a timely and thus clinical useful manner.

## Conclusion

This study established a moderate and positive correlation between ETCO2 and AMSA in a pig model of cardiac arrest. These findings provide the physiological basis for developing and testing a novel noninvasive method that utilizes either ETCO2 alone or the combination of ETCO2 and AMSA to help predict when the first defibrillation shock should be delivered in patients in cardiac arrest undergoing CPR in the field.

## Conflict of Interest

Drs. Metzger and Berger are employed by Zoll, the manufacturer of the ResQPOD^TM^ and of the ResQPUMP™. Dr. Lurie is a consultant to Zoll and the inventor of the ResQPOD^TM^ and of the ResQPUMP™. Other authors have no conflict of interest.

## References

[phy213401-bib-0001] Burnett, A. M. , N. Segal , J. G. Salzman , M. S. McKnite , and R. J. Frascone . 2012 Potential negative effects of epinephrine on carotid blood flow and ETCO2 during active compression‐decompression CPR utilizing an impedance threshold device. Resuscitation 83:1021–1024. 22445865 10.1016/j.resuscitation.2012.03.018

[phy213401-bib-0002] Callaham, M. , and C. Barton . 1990 Prediction of outcome of cardiopulmonary resuscitation from end‐tidal carbon dioxide concentration. Crit. Care Med. 18:358–362. 2108000 10.1097/00003246-199004000-00002

[phy213401-bib-0003] Cantineau, J. P. , Y. Lambert , P. Merckx , P. Reynaud , F. Porte , C. Bertrand , et al. 1996 End‐tidal carbon dioxide during cardiopulmonary resuscitation in humans presenting mostly with asystole: a predictor of outcome. Crit. Care Med. 24:791–796. 8706455 10.1097/00003246-199605000-00011

[phy213401-bib-0004] Falk, J. L. , E. C. Rackow , and M. H. Weil . 1988 End‐tidal carbon dioxide concentration during cardiopulmonary resuscitation. N. Engl. J. Med. 318:607–611. 3125432 10.1056/NEJM198803103181005

[phy213401-bib-0005] Grmec, S. , and P. Klemen . 2001 Does the end‐tidal carbon dioxide (EtCO2) concentration have prognostic value during out‐of‐hospital cardiac arrest? Eur. J. Emerg. Med. 8:263–269. 11785591 10.1097/00063110-200112000-00003

[phy213401-bib-0006] Gudipati, C. V. , M. H. Weil , J. Bisera , H. G. Deshmukh , and E. C. Rackow . 1988 Expired carbon dioxide: a noninvasive monitor of cardiopulmonary resuscitation. Circulation 77:234–239. 3121209 10.1161/01.cir.77.1.234

[phy213401-bib-0007] Hatlestad, D. 2004 Capnography as a predictor of the return of spontaneous circulation. Emerg. Med. Serv. 33; quiz 115:75–80. 15368978

[phy213401-bib-0008] He, M. , Y. Lu , L. Zhang , H. Zhang , Y. Gong , and Y. Li . 2016 Combining amplitude spectrum area with previous shock information using neural networks improves prediction performance of defibrillation outcome for subsequent shocks in out‐of‐hospital cardiac arrest patients. PLoS ONE 11:e0149115. 26863222 10.1371/journal.pone.0149115 PMC4749245

[phy213401-bib-0009] Jacobs, I. , K. Sunde , C. D. Deakin , M. F. Hazinski , R. E. Kerber , R. W. Koster , et al. 2010 Part 6: defibrillation: 2010 International Consensus on Cardiopulmonary Resuscitation and Emergency Cardiovascular Care Science With Treatment Recommendations. Circulation 122:S325–S337. 20956254 10.1161/CIRCULATIONAHA.110.971010

[phy213401-bib-0010] Levine, R. L. , M. A. Wayne , and C. C. Miller . 1997 End‐tidal carbon dioxide and outcome of out‐of‐hospital cardiac arrest. N. Engl. J. Med. 337:301–306. 9233867 10.1056/NEJM199707313370503

[phy213401-bib-0011] Lewis, L. M. , J. Stothert , J. Standeven , B. Chandel , M. Kurtz , and J. Fortney . 1992 Correlation of end‐tidal CO_2_ to cerebral perfusion during CPR. Ann. Emerg. Med. 21:1131–1134. 1514728 10.1016/s0196-0644(05)80658-4

[phy213401-bib-0012] Lurie, K. G. , P. Coffeen , J. Shultz , S. McKnite , B. Detloff , and K. Mulligan . 1995 Improving active compression‐decompression cardiopulmonary resuscitation with an inspiratory impedance valve. Circulation 91:1629–1632. 7882467 10.1161/01.cir.91.6.1629

[phy213401-bib-0013] Lurie, K. G. , E. C. Nemergut , D. Yannopoulos , and M. Sweeney . 2016 The Physiology of Cardiopulmonary Resuscitation. Anesth. Analg. 122:767–783. 26562060 10.1213/ANE.0000000000000926

[phy213401-bib-0014] Neurauter, A. , T. Eftestol , J. Kramer‐Johansen , B. S. Abella , V. Wenzel , K. H. Lindner , et al. 2008 Improving countershock success prediction during cardiopulmonary resuscitation using ventricular fibrillation features from higher ECG frequency bands. Resuscitation 79:453–459. 18954929 10.1016/j.resuscitation.2008.07.024

[phy213401-bib-0015] Noc, M. , M. H. Weil , R. J. Gazmuri , S. Sun , J. Biscera , and W. Tang . 1994 Ventricular fibrillation voltage as a monitor of the effectiveness of cardiopulmonary resuscitation. J. Lab. Clin. Med. 124:421–426. 8083585

[phy213401-bib-0016] Paradis, N. A. , G. B. Martin , E. P. Rivers , M. G. Goetting , T. J. Appleton , M. Feingold , et al. 1990 Coronary perfusion pressure and the return of spontaneous circulation in human cardiopulmonary resuscitation. JAMA 263:1106–1113. 2386557

[phy213401-bib-0017] von Planta, M. , I. von Planta , M. H. Weil , S. Bruno , J. Bisera , and E. C. Rackow . 1989 End tidal carbon dioxide as an haemodynamic determinant of cardiopulmonary resuscitation in the rat. Cardiovasc. Res. 23:364–368. 2512010 10.1093/cvr/23.4.364

[phy213401-bib-0018] Povoas, H. P. , and J. Bisera . 2000 Electrocardiographic waveform analysis for predicting the success of defibrillation. Crit. Care Med. 28:N210–N211. 11098949 10.1097/00003246-200011001-00010

[phy213401-bib-0019] Povoas, H. P. , M. H. Weil , W. Tang , J. Bisera , K. Klouche , and A. Barbatsis . 2002 Predicting the success of defibrillation by electrocardiographic analysis. Resuscitation 53:77–82. 11947983 10.1016/s0300-9572(01)00488-9

[phy213401-bib-0020] Ristagno, G. , A. Gullo , G. Berlot , U. Lucangelo , E. Geheb , and J. Bisera . 2008 Prediction of successful defibrillation in human victims of out‐of‐hospital cardiac arrest: a retrospective electrocardiographic analysis. Anaesth. Intensive Care 36:46–50. 18326131 10.1177/0310057X0803600108

[phy213401-bib-0021] Ristagno, G. , Y. Li , F. Fumagalli , A. Finzi , and W. Quan . 2013 Amplitude spectrum area to guide resuscitation‐a retrospective analysis during out‐of‐hospital cardiopulmonary resuscitation in 609 patients with ventricular fibrillation cardiac arrest. Resuscitation 84:1697–1703. 24005007 10.1016/j.resuscitation.2013.08.017

[phy213401-bib-0022] Ristagno, G. , T. Mauri , G. Cesana , Y. Li , A. Finzi , F. Fumagalli , et al., Azienda Regionale Emergenza Urgenza Research G ; 2015 Amplitude spectrum area to guide defibrillation: a validation on 1617 patients with ventricular fibrillation. Circulation 131:478–487. 25466976 10.1161/CIRCULATIONAHA.114.010989

[phy213401-bib-0023] Sanders, A. B. , M. Atlas , G. A. Ewy , K. B. Kern , and S. Bragg . 1985 Expired PCO2 as an index of coronary perfusion pressure. Am. J. Emerg. Med. 3:147–149. 3918548 10.1016/0735-6757(85)90039-7

[phy213401-bib-0024] Sanders, A. B. , K. B. Kern , C. W. Otto , M. M. Milander , and G. A. Ewy . 1989 End‐tidal carbon dioxide monitoring during cardiopulmonary resuscitation. A prognostic indicator for survival. JAMA 262:1347–1351. 2761035

[phy213401-bib-0025] Schultz, J. , N. Segal , J. Kolbeck , S. McKnite , E. Caldwell , and D. Yannopoulos . 2012 Sodium nitroprusside enhanced cardiopulmonary resuscitation (SNPeCPR) improves vital organ perfusion pressures and carotid blood flow in a porcine model of cardiac arrest. Resuscitation 83:374–377. 21864483 10.1016/j.resuscitation.2011.07.038 PMC3244558

[phy213401-bib-0026] Trevino, R. P. , J. Bisera , M. H. Weil , E. C. Rackow , and W. G. Grundler . 1985 End‐tidal CO_2_ as a guide to successful cardiopulmonary resuscitation: a preliminary report. Crit. Care Med. 13:910–911. 3931980 10.1097/00003246-198511000-00012

[phy213401-bib-0027] Weaver, W. D. , L. A. Cobb , D. Dennis , R. Ray , A. P. Hallstrom , and M. K. Copass . 1985 Amplitude of ventricular fibrillation waveform and outcome after cardiac arrest. Ann. Intern. Med. 102:53–55. 3966746 10.7326/0003-4819-102-1-53

[phy213401-bib-0028] Weil, M. H. , J. Bisera , R. P. Trevino , and E. C. Rackow . 1985 Cardiac output and end‐tidal carbon dioxide. Crit. Care Med. 13:907–909. 3931979 10.1097/00003246-198511000-00011

[phy213401-bib-0029] Xie, J. , M. H. Weil , S. Sun , W. Tang , Y. Sato , X. Jin , et al. 1997 High‐energy defibrillation increases the severity of postresuscitation myocardial dysfunction. Circulation 96:683–688. 9244243 10.1161/01.cir.96.2.683

